# Development and validation of a clinical prediction model for the risk of distal metastasis in intrahepatic cholangiocarcinoma: a real-world study

**DOI:** 10.1186/s12876-023-03084-9

**Published:** 2024-01-02

**Authors:** Caixia Fang, Chan Xu, Xiaodong Jia, Xiaoping Li, Chengliang Yin, Xiaojuan Xing, Wenle Li, Zhenyun Wang

**Affiliations:** 1 Pharmacy Department, Clinical Drug Research Center, Qingyang People’s Hospital, Qingyang, China; 2 State Key Laboratory of MolecularVaccinology and Molecular Diagnostics & Center for Molecular Imaging and Translational Medicine, School of Public Health, Xiamen University, Xiamen, 361102 China; 3 Comprehensive Liver Cancer Center, The Fifth Medical Center of PLA General Hospital, Beijing, China; 4 Shulan International Medical College, Zhejiang Shuren University, Hangzhou, China; 5 Faculty of Medicine, Macau University of Science and Technology, Macau, China; 6 Department of Neurology, Qingyang People’s Hospital, Qingyang, China; 7 Urology Department of Qingyang People’s Hospital, Qingyang, China

**Keywords:** Cholangiocarcinoma, Distal metastasis, Prediction model, Nomogram, Risk factor

## Abstract

**Background:**

Cholangiocarcinoma (CCA) is a highly malignant and easily metastatic bile duct tumor with poor prognosis. We aimed at studying the associated risk factors affecting distal metastasis of CCA and using nomogram to guide clinicians in predicting distal metastasis of CCA.

**Methods:**

Based on inclusion and exclusion criteria, 345 patients with CCA were selected from the Fifth Medical Center of Chinese PLA General Hospital and were divided into distal metastases (*N =* 21) and non-distal metastases (*N =* 324). LASSO regression models were used to screen for relevant parameters and to compare basic clinical information between the two groups of patients. Risk factors for distal metastasis were identified based on the results of univariate and multivariate logistic regression analyses. The nomogram was established based on the results of multivariate logistic regression, and we drawn the corresponding correlation heat map. The predictive accuracy of the nomogram was evaluated by receiver operating characteristic (ROC) curves and calibration plots. The utility of the model in clinical applications was illustrated by applying decision curve analysis (DCA), and overall survival(OS) analysis was performed using the method of Kaplan-meier.

**Results:**

This study identified 4 independent risk factors for distal metastasis of CCA, including CA199, cholesterol, hypertension and margin invasion, and developed the nomogram based on this. The result of validation showed that the model had significant accuracy for diagnosis with the area under ROC (AUC) of 0.882 (95% CI: 0.843-0.914). Calibration plots and DCA showed that the model had high clinical utility.

**Conclusions:**

This study established and validated a model of nomogram for predicting distal metastasis in patients with CCA. Based on this, it could guide clinicians to make better decisions and provide more accurate prognosis and treatment for patients with CCA.

## Introduction

Cholangiocarcinoma (CCA) is the second most common primary hepatobiliary malignancy in the world and the most common malignancy of biliary tract [1–3]. It is second only to hepatocellular carcinoma in terms of incidence, and is characterized by extremely insidious and highly aggressive onset. The highest incidence rates are in Asia, particularly in South Korea, Thailand, and Japan, and the incidence of ICC and ECC has increased in most countries between 1993 and 2012 [4]. Patients can only have a chance of long-term survival if they undergo R0 surgical resection, but most patients are already at an advanced stage of the disease or have developed metastases of distal organ when they are diagnosed in outpatient clinics, and are often deprived of the chance of operation, so the overall prognosis of CCA patients is extremely poor, with an overall survival(OS) rate of less than 10% at 5 years [1, 5–8]. Additionally, it’s important to note that the risk factors for CCA include chronic liver diseases, such as hepatitis B and C, liver flukes infestation, and certain genetic disorders, which further complicate its global impact and the need for diverse research approaches [9]. On the other hand, even if patients receive surgical treatment, the high recurrence rate after surgery leads to a threat to long-term survival, and some reports show that the OS rate of CCA patients at 5 years after surgery is about 20-35% [10]. In recent decades, the incidence of ICC has gradually increased worldwide [11–14]. Therefore, studies on the metastasis of CCA are really significant for the diagnosis and treatment of CCA, and will play an important role in the prevention and treatment of CCA.

In recent years, the establishment of statistical prediction models has become a major hot spot in clinical oncology research. Nomogram have been widely used as a highly individualized visual prediction tool for clinical prognosis prediction and decision making [15–19]. The main feature of nomogram is the ability to create a convenient visual graph that can accurately calculate clinical survival probabilities based on the parameters of a statistical regression model [20–22]. Based on the lack of good methods for predicting distal metastasis in CCA, we constructed a nomogram for predicting distal metastasis in CCA patients, hoping to improve the prediction of distal metastasis.

## Materials and methods

### Collection of clinical information

In this study, the clinicopathological data of patients diagnosed with CCA in the department of hepatobiliary surgery, the Fifth Medical Center of Chinese PLA General Hospital were collected from July 2007 to July 2019 and patients were screened according to the following inclusion/exclusion criteria: 1) Patients with clinically and pathologically confirmed primary cholangiocarcinoma. 2) Patients not lost to follow-up. 3) Clear description of tumor invasion. 4) Complete baseline information. 5) Clear treatment information. Of the 345 cases, 21 cases were distal metastases, and 324 cases were non-distal metastases. Detailed information on patients’ age, tumor stage and grade were also collected. The tumor stage was determined based on the TNM staging system, and the grade was based on histopathological evaluation. The patient recruitment process involved outreach through our hospital’s database, where eligible patients were identified and contacted. The inclusion criteria for the study were defined as any patient with a diagnosis of primary CCA, and exclusion criteria included secondary or metastatic cholangiocarcinoma, incomplete medical records, and refusal to participate in the study.

The information contained relevant information on patients’ gender, survival status, survival time, AFP level, CA-199 level, hypertension, cholecystectomy, Fe, Glucose (Glu), tumor number, cholesterol, lymph nodes, surgical margin invasion, bile duct dilatation, monocyte, phosphorus (P), Golgi apparatus protein (GP) and degree of differentiation.

Tumor invasion was determined by a combination of imaging and intraoperative observation. Intraoperative pathology has been determined as the gold standard, and if there is no pathology, there is imaging examination and the judgment of professional imaging doctors.

### Parameter filter

LASSO regression models were used for screening of relevant parameters. For baseline information of patients, T-test was used for comparison as well as Chi-square test. We further analyzed the factors affecting distal metastasis in patients with CCA by univariate logistic regression. The independent risk factors associated with distal metastasis in patients with CCA were then analyzed by multivariate logistic regression. The selection of the LASSO regression model was based on its effectiveness in handling high-dimensional data and its ability to enhance model accuracy by reducing overfitting. This model was validated using a bootstrapping method to ensure its reliability [23].

### Model construction and validation

Based on the results of parameter screening, we plotted the correlation heat map. And based on the results of multivariate logistic regression analysis, we constructed the correlation column line plot model and plotted the receiver operating characteristic (ROC) curve and calculated the area under ROC (AUC) to evaluate the accuracy of the prediction of the nomogram. The predictive ability of the distal metastasis prediction model for CCA was verified by calibration plots. Decision curve analysis (DCA) was applied to compare the net benefit and evaluate the clinical utility of the nomogram. Model validation was performed using a separate validation cohort to assess the generalizability and robustness of the model. The calibration of the model was assessed using calibration plots, and the DCA was used to evaluate the clinical usefulness of the model in decision-making processes [24].

### Statistical analysis

Continuous variables were expressed using mean ± standard deviation (Mean ± SD) and categorical variables were expressed using frequency and percentages. Shapiro-Wilk test, T-test, Chi-square test, univariate and multivariate logistic regression analyses were performed using SPSS 26.0 software (SPSS Inc., Chicago, USA).R language (version 4.0.5) was used to plot the corresponding correlation heat maps, nomogram, ROC curves, calibration plots,DCA curves and assess the overall survival(OS) of patients by using the Kaplan-Meier method. *P* < 0.05 was considered as a significant difference. The use of SPSS and R language was chosen for their robust statistical analysis capabilities and flexibility in handling complex medical data. These tools allowed for comprehensive data analysis, including the construction of nomograms and ROC curves, to ensure the accuracy and reliability of our results.

## Results

### Parameter screening and population baseline

**Fig. 1:**
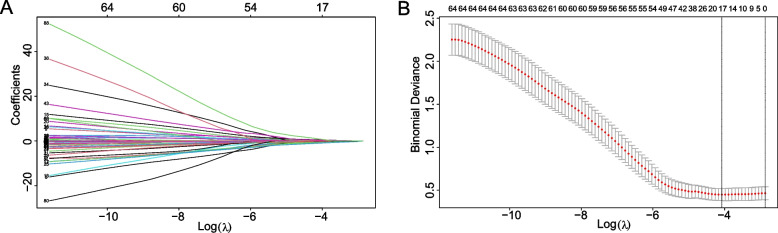
The LASSO regression used for parameter screening. **A** Each curve represents a variable. **B** 10-fold cross-validation to adjust parameter selection in the LASSO model

A total of 345 patients, including 21 patients with distal metastases and 324 patients with non-distal metastases, were included in this study. Seventeen relevant parameters were screened using the LASSO regression model, including hypertension, cholecystectomy, tumor number, bile duct dilatation, lymph nodes, degree of differentiation, margin invasion, CA199, boundary, gender, monocyte, P, Fe, Glu, cholesterol, GP and AFP. Ten parameters were obtained after further screening, including AFP, bile duct dilatation, gender, degree of differentiation, hypertension, CA199, P, tumor number, cholesterol and margin invasion (Fig. 1a, b).

**Fig. 2:**
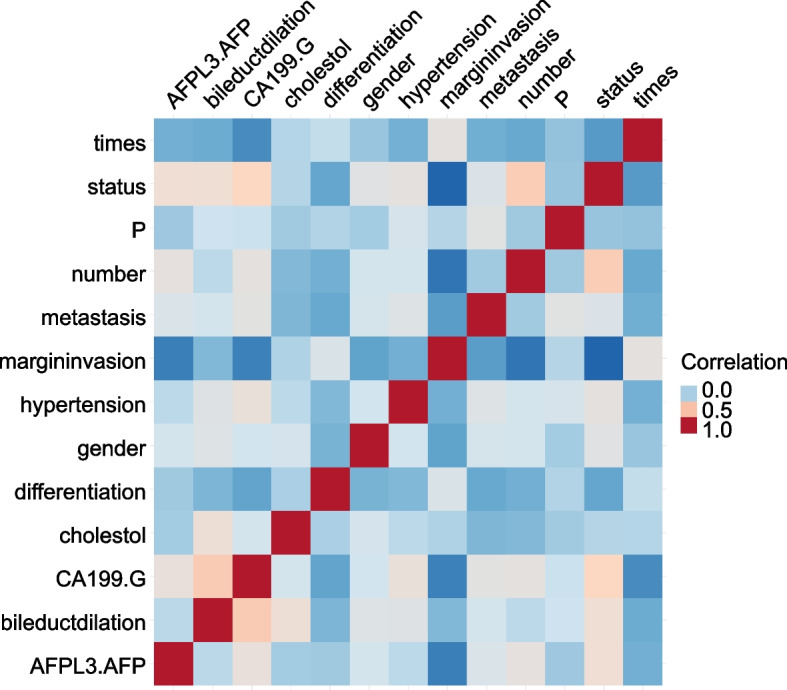
The correlation heat map of the parameters

We analyzed the baseline information of the two groups (distal metastases or not) by relevant statistical methods and the results showed that the two groups showed statistical differences in status, survival time, AFP, bile duct dilatation, gender, degree of differentiation, hypertension, CA199, P, cholesterol and margin invasion (Table 1, *p* < 0.05). No significant difference was seen in the number of tumors (Table 1, *p* > 0.05). Correlation analysis of the parameters was performed and the corresponding correlation heat map was drawn (Fig. 2).

**Table 1:** Baseline of distal metastasis in a patient with cholangiocarcinoma

Characteristics	level	No(***N =*** 324)	Yes(***N =*** 21)	p
**status (%)**	alive	178 (54.9)	4 (19.0)	0.003
	dead	146 (45.1)	17 (81.0)	
**times (mean (SD))**	NA	16.46 (12.92)	7.99 (6.20)	0.003
**AFPL3.AFP (mean (SD))**	NA	0.10 (0.09)	0.17 (0.11)	0.002
**Bile duct dilation (%)**	no	260 (80.2)	12 (57.1)	0.025
	yes	64 (19.8)	9 (42.9)	
**gender (%)**	female	62 (19.1)	9 (42.9)	0.02
	male	262 (80.9)	12 (57.1)	
**differentiation (%)**	moderately differentiated	262 (80.9)	11 (52.4)	0.002
	Poorly differentiated	55 (17.0)	10 (47.6)	
	well differentiated	7 (2.2)	0 (0.0)	
**hypertension (%)**	no	287 (88.6)	13 (61.9)	0.001
	yes	37 (11.4)	8 (38.1)	
**CA199.G (%)**	<39	202 (62.3)	2 (9.5)	<0.001
	>1000	58 (17.9)	8 (38.1)	
	39-1000	64 (19.8)	11 (52.4)	
**P (mean (SD))**	NA	1.62 (4.77)	64.91 (286.00)	<0.001
**number (%)**	Double	76 (23.5)	4 (19.0)	0.844
	single	248 (76.5)	17 (81.0)	
**cholestol (mean (SD))**	NA	4.27 (1.29)	3.63 (0.84)	0.026
**Margin invasion (%)**	no	136 (42.0)	19 (90.5)	<0.001
	yes	188 (58.0)	2 (9.5)

### Univariate and multivariate logistic regression analysis

Univariate and multivariate logistic regression analyses were performed on the parameters screened by LASSO regression. Univariate logistic regression analysis showed that AFP (OR = 62.9, 95%CI =2.94-1347.14, *p* < 0.05), bile duct dilatation (yes OR = 3.05, 95%CI = 1.23-7.54, *p* < 0.05), CA199(39-1000 OR = 17.36, 95%CI = 3.75-80.38, *p* < 0.001; > 1000 OR = 13.93, 95%CI = 2.88-67.42, *p* = 0.001), cholesterol (OR = 0.48, 95% CI = 0.28-0.83, *p* < 0.05), degree of differentiation (poorly differentiation OR = 4.33, 95% CI = 1.75-10.7, *p* = 0.001), gender (male OR = 0.32, 95%CI = 0.13-0.78, *p* < 0.05), hypertension (yes OR = 4.77, 95%CI = 1.86-12.28, *p* = 0.001), and margin invasion (yes OR = 0.08, 95%CI = 0.02-0.33, *p* = 0.001) were significant factors for distal metastasis in patients with CCA(Tab. 2). Further multivariate logistic regression analysis showed that CA199 (39-1000 OR = 9.81, 95%CI = 1.81-53.15, *p* < 0.05), cholesterol (OR = 0.39, 95%CI = 0.2-0.77, *p* < 0.05), hypertension (yes OR = 3.78, 95%CI = 1.19-12.02, *p* < 0.05), and margin invasion (yes OR = 0.1, 95%CI = 0.02-0.59, *p* < 0.05) were independent risk factors for distal metastasis in patients with CCA (Table 2).

**Table 2:** Univariate and multivariable logistics regression

Characteristics	Univariate logistics regression			Multivariable logistics regression		
	OR	95%CI	P	OR	95%CI	P
**AFPL3.AFP**	62.9	2.94-1347.14	0.008	16.54	0.3-921.16	0.171
**Bile duct dilation**						
** no**	Ref	Ref	Ref	Ref	Ref	Ref
**yes**	3.05	1.23-7.54	0.016	2.96	0.86-10.21	0.085
**CA199.G**						
**<39**	Ref	Ref	Ref	Ref	Ref	Ref
**>1000**	13.93	2.88-67.42	0.001	3.97	0.69-22.75	0.121
**39-1000**	17.36	3.75-80.38	< 0.001	9.81	1.81-53.15	0.008
**Cholestol**	0.48	0.28-0.83	0.008	0.39	0.2-0.77	0.006
**Differentiation**						
**moderately differentiated**	Ref	Ref	Ref	Ref	Ref	Ref
**poorly differentiated**	4.33	1.75-10.7	0.001	2.39	0.82-7.02	0.112
**well differentiated**	0	0-Inf	0.992	0	0-Inf	0.991
**Gender**						
**female**	Ref	Ref	Ref	Ref	Ref	Ref
**male**	0.32	0.13-0.78	0.013	0.34	0.11-1.03	0.057
**P**	1.02	0.96-1.08	0.449	NA	NA	NA
**Hypertension**						
**no**	Ref	Ref	Ref	Ref	Ref	Ref
**yes**	4.77	1.86-12.28	0.001	3.78	1.19-12.02	0.024
**Margin invasion**						
**no**	Ref	Ref	Ref	Ref	Ref	Ref
**yes**	0.08	0.02-0.33	0.001	0.1	0.02-0.59	0.011
**Number**						
**double**	Ref	Ref	Ref	Ref	Ref	Ref
**single**	1.3	0.43-3.99	0.644	NA	NA	NA

**Fig. 3:**
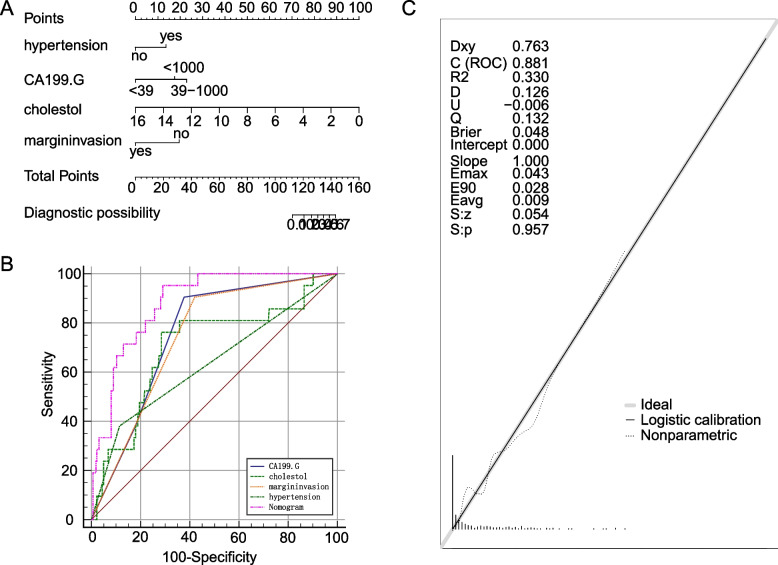
**A** The nomogram for the risk of distal metastasis in patients with CCA. **B** ROC of the nomogram. **C** The calibration plots of the model

To predict the risk of distal metastasis in patients with CCA, we created a nomogram based on multivariate logistic regression results (Fig. 3a). We found that cholesterol had the greatest effect on distal metastasis, and hypertension had the least effect on distal metastasis (Fig. 3b). the AUC was 0.882 (95%CI = 0.843-0.914,Cuf-off = 0.6623) (Table 3), indicating that the nomogram was more accurate in assessing distal metastasis in patients with CCA and had higher accuracy compared with univariate prediction. Then the calibration curve showed a better match between actual and predicted occurrence (Fig. 3c).

**Table 3:** The AUC values of the nomogram

Variable	AUC	95% CI
**CA199.G**	0.755	0.706 to 0.799
**cholestol**	0.703	0.652 to 0.751
**Margin invasion**	0.743	0.693 to 0.788
**hypertension**	0.633	0.580 to 0.684
**Nomogram**	0.882	0.843 to 0.914

### Clinical applications of the nomogram

**Fig. 4:**
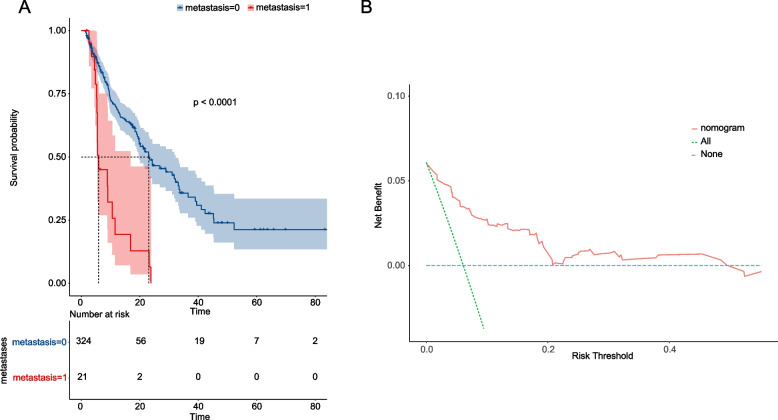
**A** The Kaplan-Meier overall survival (OS) analysis of patients with distal metastases or not. **B** The DCA plots of nomogram for the risk of distal metastasis in patients with CCA. The red curve shows the number of people classified as positive of each threshold probability, and the green one shows the number of true positives

Overall survival (OS) Kaplan-Meier survival curves were plotted for the two groups of patients who developed distal metastases or not (Fig. 4a). The results showed that the survival of patients with distal metastases was significantly lower in those who developed distal metastases compared to those who did not (*P* < 0.0001). Meanwhile, we plotted DCA plots for assessing the clinical utility of the model, which showed good clinical utility in predicting distal metastasis in patients with CCA (Fig. 4b).

## Discussion

CCA is a malignant tumor originating from the epithelial cells of the bile duct system, which accounts for 10-15% of primary liver cancer, but is more malignant [12, 25–28]. CCA is prone to intrahepatic and lymph node metastasis and lacks effective early diagnostic indicators, so patients are diagnosed at an advanced stage [29–31]. In recent years, the establishment of statistical prediction models has become a hot topic in clinical research on cancer [32, 33]. We analyzed the clinical data of 345 patients with CCA to explore the factors that may affect the prognosis and to establish a distal metastasis prediction model to effectively evaluate the prognosis of patients. This model can aid clinicians in identifying high-risk patients early, allowing for more personalized and timely interventions, potentially improving the overall prognosis. At the same time, this study is the first to date to focus on predicting the distal risk of CCA.

At present, the diagnosis of CCA is mainly a comprehensive diagnosis combining medical history, imaging diagnosis and serological diagnosis; imaging diagnosis is mainly based on CT and MRI [34], serological diagnosis is mainly CA199 and carcinoembryonic antigen, and the sensitivity and specificity of CA199 for the diagnosis of CCA are 62 and 63% respectively [31, 35, 36]. Meanwhile, preoperative concentration of CA199 greater than 100 U/ml significantly shortens the CCA patients’ postoperative tumor-free survival time [37]. Another study pointed out that preoperative serum CA199 levels higher than 135 U/ml could predict the prognosis of patients with CCA to some extent, but lacked some accuracy [38]. Our findings were similar to our predecessors. The results showed CA199 to be an independent risk factor for the prognosis of distal metastases in diagnosing CCA. These insights can be integrated into clinical decision-making processes, helping clinicians to better predict and manage the course of the disease.

Related studies have shown that residual cancer at the incisal margin was an extremely important factor affecting prognosis [39], and our study also showed that surgical margin invasion was an independent risk factor for predicting distal metastasis from CCA. Multiple infiltration and metastasis could occur early along the peribiliary lymphatics, blood vessels, perineural spaces, and sparse fibrous connective tissue. In addition, the presence of abundant lymphatics, blood vessels, nerve fibers and loose connective tissues in and around the bile ducts provided an avenue for the “jumping” growth of bile duct cancer cells [40]. Understanding these factors can enhance surgical planning and postoperative management, tailoring treatment to individual patient profiles.

Our study showed the important role of hypertension in the prediction of distal metastasis in patients with CCA. About 38% of these patients with distal metastasis had hypertension in this study, whereas only 11% of these patients with non-distal metastasis had hypertension in this study. Further studies are needed to address the mechanisms associated with hypertension and distal metastasis in CCA. This finding suggests that monitoring and managing hypertension in CCA patients could be a key aspect of their overall treatment plan.

Currently, metabolic syndrome was considered a risk factor for CCA [41–43]. Our study found that cholesterol was an important independent risk factor for the prediction of distal metastasis in patients with CCA. Although the exact role of lipids in CCA was unclear, we believed that cholesterol played a key role in the prediction of distal metastasis in patients with CCA. This observation opens avenues for exploring lipid-lowering strategies as part of the therapeutic approach for CCA.

Our study also has some limitations. This was a retrospective study with data from the single clinical center. The data lack detailed information in some patients’ conditions, such as the specifics of bile duct obstruction or dilatation, vascular invasion, and some biochemical indicators such as prealbumin and some hepatitis virus infections, making it difficult to conduct a more in-depth analysis. At the same time, it was difficult to obtain cases of intrahepatic cholangiocarcinoma, and there were subgroup imbalances in the data. In addition, asa single center clinical study, the results might have some bias.

## Conclusion

We collected and processed the clinical data of a large number of patients with CCA to establish a nomogram of distal metastasis in patients with CCA and further evaluated the model. The model is important for predicting the risk of distal metastasis in patients with CCA, providing a risk assessment tool for clinicians to grasp the overall situation of patients, and providing theoretical support for the formulation of patients treatment plans.

## Data Availability

The data used in this study can be obtained from the corresponding author on reasonable grounds.
